# Curvilinear Effects of Invasive Plants on Plant Diversity: Plant Community Invaded by *Sphagneticola trilobata*

**DOI:** 10.1371/journal.pone.0113964

**Published:** 2014-11-26

**Authors:** Shan-Shan Qi, Zhi-Cong Dai, De-Li Zhai, Si-Chong Chen, Chun-Can Si, Ping Huang, Rui-Ping Wang, Qiong-Xin Zhong, Dao-Lin Du

**Affiliations:** 1 Institute of Environment and Ecology, School of the Environment and Safety Engineering, Jiangsu University, Zhenjiang, China; 2 World Agroforestry Centre (ICRAF), Central and East Asia Office, Kunming, China; 3 Centre for Mountain Ecosystem Studies (CMES), Kunming Institute of Botany, Chinese Academy of Sciences, Kunming, China; 4 Evolution and Ecology Research Centre, School of Biological, Earth and Environmental Sciences, University of New South Wales, Sydney, Australia; 5 Department of Biological and Chemical Engineering, Jingdezhen University, Jingdezhen, China; 6 Key Laboratory of Modern Agricultural Equipment and Technology, Ministry of Education & Jiangsu Province, Jiangsu University, Zhenjiang, China; 7 College of Life Sciences, Hainan Normal University, Haikou, China; Beijing Forestry University, China

## Abstract

The effects of invasive plants on the species diversity of plant communities are controversial, showing either a positive or negative linear relationship. Based on community data collected from forty 5 m×5 m plots invaded by *Sphagneticola trilobata* in eight cities across Hainan Island, China, we found *S. trilobata* decreased plant community diversity once its cover was beyond 10%. We demonstrated that the effects of invasive/native plants on the plant diversity of communities invaded by *S. trilobata* were curvilinear. These effects, which showed peaks under different degrees of vegetation cover, appeared not only for *S. trilobata* and all invasive plants, but also for all native plants. Invasive plants primarily had negative effects on plant diversity when they became abundant at a much lower cover level (less than 35%), compared with the native plants (over 60%). Thus, it is necessary to distinguish a range for assessing the effects of plants, especially invasive plants. Our results also confirmed that the invasion intensity of invasive alien plants increased with the intensity of local economic development. We highlight and further discuss the critical importance of curvilinear effects of biological invasion to provide ideas regarding the conservation of local biodiversity and the management of invasive plants.

## Introduction

Biodiversity is important in the functional provision and stability of ecosystems [1], [2]. However, biodiversity is continuously being lost due to human disturbance [3]. For example, unprecedented land conversion plays a key role in weed invasion through destroying native plant communities and facilitating the invasion of non-indigenous plants [4]–[6]. Continual development, the expansion of urbanization, and the conversion of forest lands for agricultural development lead to increasing losses of native species [7], [8] and result in the establishment and spreading of alien inhabitants in these communities [5]. These non-indigenous inhabitants can change the structure and diversity of the plant community by suppressing subordinate species [9]. Thus, exotic species invasion is recognized as one of the most serious global threats to natural ecosystems [10]. Exotic invasions continue to crowd out native plant species and homogenize biota around the world [11], [12], with the consequences of decreasing global biodiversity [13] and impairing ecosystem functions [10], [14].

Increasing efforts are being made to elucidate the mechanisms underlying exotic invasion to find solutions for the restoration of invaded landscapes. However, the effects of invasive plants on species diversity in plant communities are still controversial [1], [15]. Furthermore, the scarcity of quantitative studies on the impacts of invasive plant species on plant communities limits our understanding of the impacts of invasion [16]–[18].

*Sphagneticola trilobata* (L.C. Rich.) Pruski (Synonym: *Wedelia trilobata* (L.) Hitchc.), a widespread notorious clonal weed that is native to tropical America, was introduced as a groundcover plant and has invaded many tropical and subtropical regions, including Hainan Island, China [19], [20]. *S. trilobata* is a fast-growing, mat-forming perennial creeping herb [21], [22]. *S. trilobata*'s dominance over grass is particularly noteworthy [19]. Once this species becomes established in new habitats, it exhibits overgrowth to form thick ground cover, crowding out and preventing the regeneration of other plant species [21]. Because of its noteworthy dominance over grass communities, *S. trilobata* has been listed as one of the 100 World's Worst Invasive Alien Species [23]. Nevertheless, no quantitative evaluations of the impact of *S. trilobata* on resident plants have been reported to date.

In the present study, we used *S. trilobata* and the associated invaded community as a model system to address the question of the possible impacts of *S. trilobata* and other invasive plants on the plant diversity of the invaded community.

## Methods

### Sample sites

**Figure 1:**
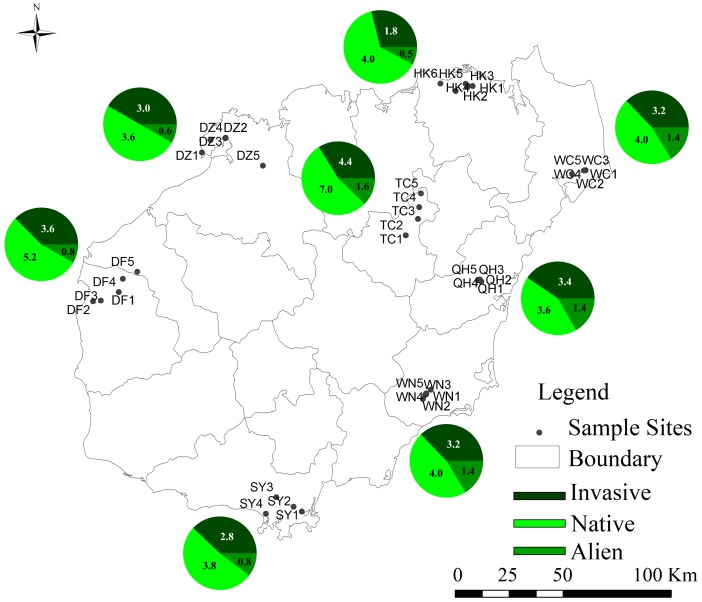
Distribution of sampling cities on Hainan Island and the average species number in each district. The pie charts show the mean number of the invasive, native, and alien non-invasive species in a 5 m×5 m plot. Dongfang (DF), Danzhou (DZ), Haikou (HK), Qionghai (QH), Sanya (SY), Tunchang (TC), Wenchang (WC), and Wanning (WN).

Field investigations were conducted on Hainan Island, China, in May 2007 to evaluate the influence of *S. trilobata* and other invasive plants on native plants and the diversity of local species. Sampling sites were selected along roads in eight cities/districts, which were evenly distributed on Hainan Island: Dongfang (DF), Danzhou (DZ), Haikou (HK), Qionghai (QH), Sanya (SY), Tunchang (TC), Wenchang (WC), and Wanning (WN) (Fig. 1). We randomly established five sites on public land in which *S. trilobata* appeared at each city (except for Haikou, where there were six sites, and Sanya, with four sites). Each site was separated from the others by a distance of at least 500 m. Then, one plot (5 m×5 m) was randomly sampled from each site. In total, 40 plots, containing nine species on average, were surveyed.

All of the vascular plant species present in each plot were recorded, and the coverage of each species in the sampled plots was estimated using a subdivided quadrat method [24], [25]. The compositions of the plant communities were distinguished as native, alien non-invasive, and invasive plants. Native plants were identified according to the Chinese Virtual Herbarium (CVH, http://www.cvh.org.cn/cms/), and non-native plants were identified as invasive or alien non-invasive (alien for short) plants according to the Database of Invasive Alien Species in China (DIASC, http://www.chinaias.cn/wjPart).

### Community diversity metrics

Shannon-Wiener's species diversity index (*H′*) [26], Simpson's dominance index (*D′*) [27], and Shannon-Wiener's evenness index (*J*′) [28] were used to test the community effects of the compositions (*S. trilobata*, all invasive plants and native plants) of the invaded communities. All of these indices were based on the cover of a given species in the sampled plots [24]. The equations for calculating the above indices are follows: (1) *H′* = -Σ[(*P_i_*) ln(*P*_i_)]; (2) *D′* = 1/Σ(*P_i_*)^2^; and (3) *J*′ = *H*′/(ln*S*). *P*_i_ is the ratio of the cover of species i to the total cover of all species in each plot, and *S* is the number of species in each plot [29].

### Statistical analyses

To evaluate the quantitative effects of the community compositions (*S. trilobata*, all invasive plants and all native plants) on the plant diversity index (*H′*), community dominance index (*D′*) and community evenness index (*J*′) of the invaded communities, the cover values for *S. trilobata*, all invasive plants and native plants were classified into four grades [30]: I-0∼25%, II-26∼50%, III-51∼75%, and IV-76∼100%, corresponding to slight, moderate, excess, and severe invasion phases, respectively. All data were logistic transformed if necessary to meet assumptions of normality and homoscedasticity before analysis. We applied two-way analysis of variance (ANOVA) using a generalized linear model (GLM), with the plant cover grades (I, II, III and IV) and sampling cities as the grouping factors, and we employed Duncan's multiple-range test (α = 0.05) to compare means among the four grades of cover of invasive *S. trilobata*, all invasive plants (including *S. trilobata*) and native plants among cities. A quadratic non-linear regression model was used to detect the relationships between the plant diversity of the community and the cover of *S. trilobata*, all invasive plants and all native plants.

To test the impacts of human disturbance on the distribution of *S. trilobata*, all invasive plants and native plants, data on geographical locations (including longitude, latitude and altitude) and the economy (including farming, forestry, grazing output and total economic output) were analyzed via Pearson correlation analysis. The total economic output included farming, forestry and grazing in each sampled city in 2006 [31], which would significantly change the local vegetation. Then, to visualize the plant cover distribution pattern, as affected by geography and the economy, we constructed Free Energy Landscape graphs using the data on the average plant cover (*S. trilobata*, all invasive plants and native plants), geographical location, and total economic output across the eight sampled cities.

All data were analyzed using SAS statistical software (v9.1) [32] and visualized using SigmaPlot Version 11.

## Results

### Relationships between invasive and native plants

**Figure 2:**
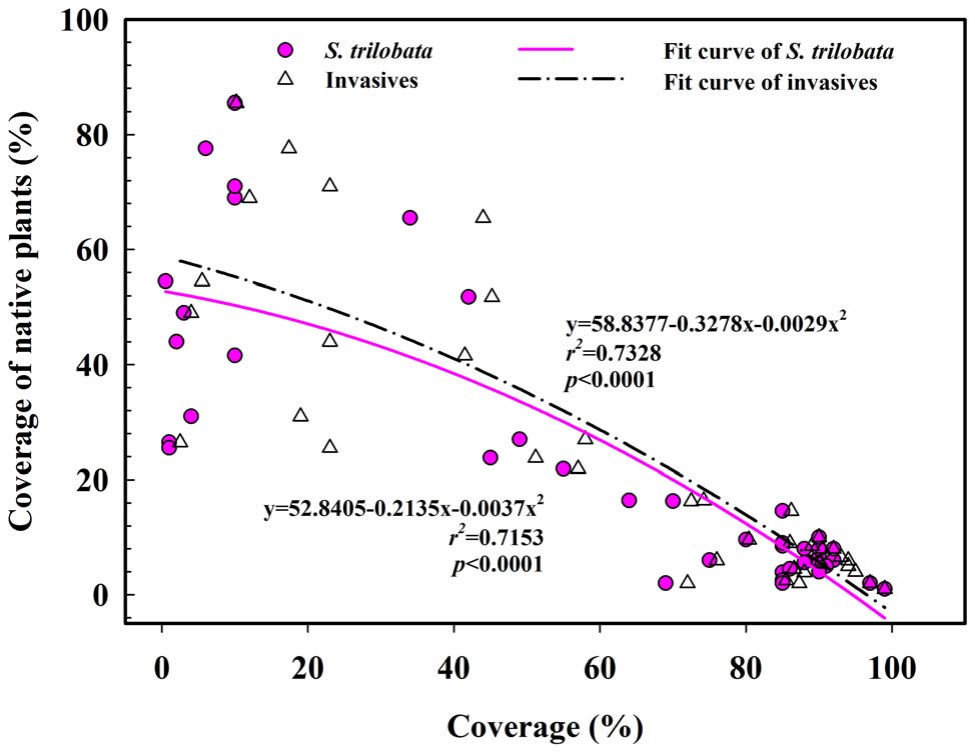
Relationships between the coverage of native plants and both the coverage of *S. trilobata* and all invasive plants.

The quadratic non-linear regression analysis indicated that the cover of invasive *S. trilobata* (*r* ^2^ = 0.72, *p*<0.0001) and all invasive plants (*r* ^2^ = 0.73, *p*<0.0001) both showed a significant negative correlation with the cover of native plants (Fig. 2).

### Effects of the species composition on plant diversity

The maximum number of species appeared at Wenchang (WC) and Wanning (WN), while the minimum number of species was recorded at Haikou (HK) (Fig. 1). Finally, a total of 65 plant species (including *S. trilobata* and twelve other invasive species, ten alien species, and 42 native species; Table S1) were identified in the sampled plots. The cover values obtained for *S. trilobata*, the other invasive plants, the alien non-invasive plants, and the native plants ranged from 0.5% to 99%, 0% to 31.5%, 0% to 31%, and 1% to 85.5%, respectively.

**Figure 3:**
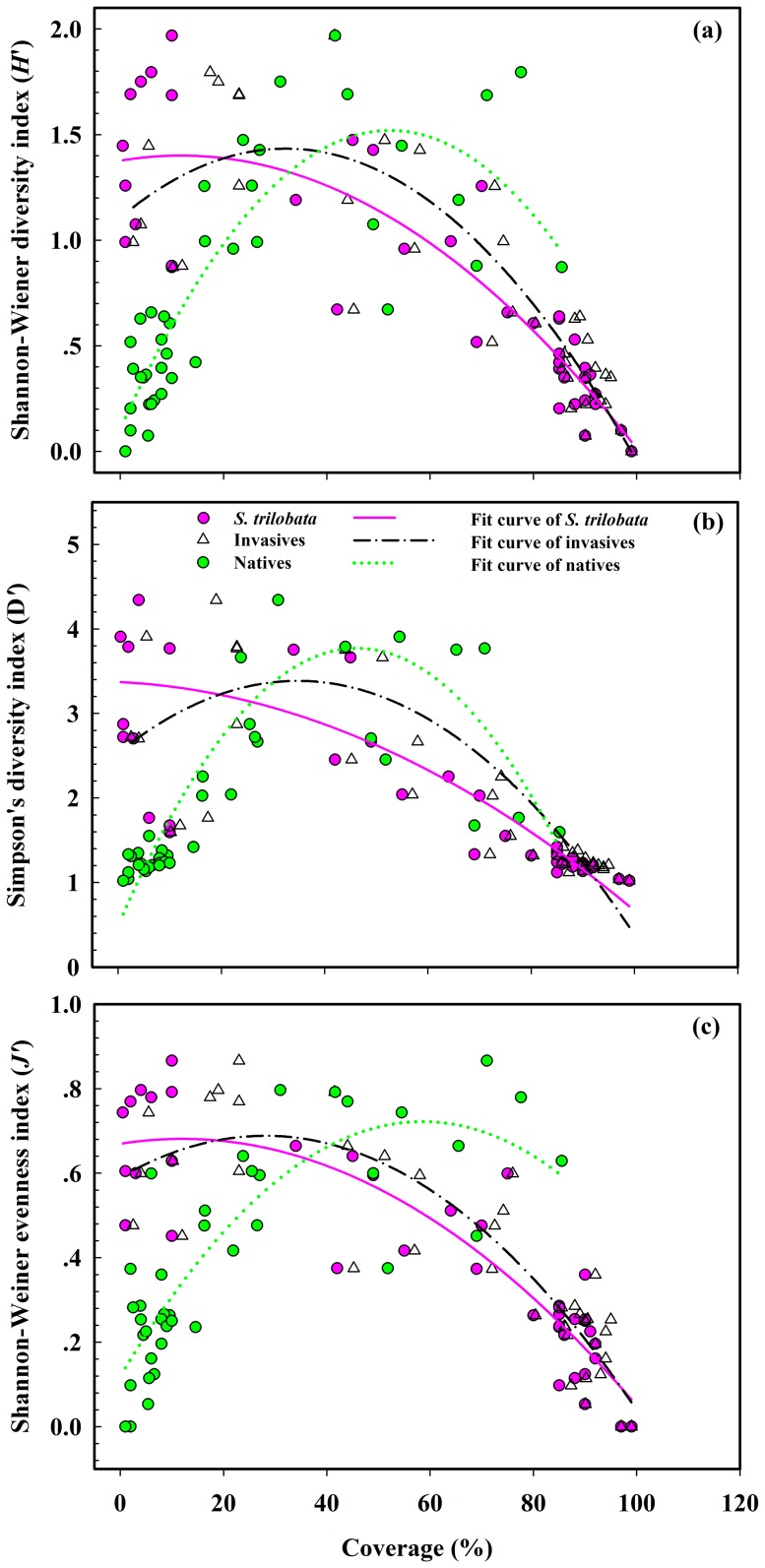
Relationships between community biodiversity indices and coverage within the community composition (*S. trilobata*, all invasive plants, and all native plants). (a): Shannon-Wiener's species diversity index, *H*′ = -Σ[(*P_i_*) ln(*P*_i_)]; (b): Simpson's dominance index, *D*′ = 1/Σ(*P_i_*)^2^; (c): Shannon-Wiener's evenness index, *J*′ = *H*'/(ln*S*).

The changes in plant community diversity in response to the species composition (*S. trilobata*, all invasive plants and all native plants) were similar, exhibiting a quadratic pattern, but did not present a simple linear relationship (Fig. 3a-c; Table 1). The cover of both all invasive plants and all native plants was a relatively stable predictor of peak plant diversity (species diversity, *H*′; species dominance, *D*′; and species evenness, *J*′) in the community invaded by *S. trilobata* (approx. 35% for invasive plants and 50% for native plants; Table 1). For *S. trilobata*, the peak plant diversity appeared when the cover was less than 10% (Table 1).

**Table 1:** Relationships between coverage within the community composition and community biodiversity indices.

Index	Compositions	Quadratic regression equation	x_ymax_(%)	y_max_	*r* ^2^	*F*	*p*
Shannon-Wiener's Species Diversity Index (*H*′)	*S. trilobata*	y = 1.3762+0.0042x-0.0002x^2^	10.5	1.4	0.77	62.94	<0.0001
	Invasives	y = 1.1069+0.0204x-0.0003x^2^	34.0	1.5	0.77	62.74	<0.0001
	Natives	y = 0.106+0.0541x-0.0005x^2^	54.1	1.6	0.71	44.42	<0.0001
Simpson's Dominance Index (*D*′)	*S. trilobata*	y = 0.6695+0.002x-8.1546*e^−0.005^x^2^	0.0001	0.7	0.81	78.67	<0.0001
	Invasives	y = 0.5898+0.007x-0.0001x^2^	35.0	0.7	0.80	74.38	<0.0001
	Natives	y = 0.1188+0.0206x-0.0002x^2^	51.5	0.6	0.70	42.37	<0.0001
Shannon-Wiener's Evenness Index (*J*′)	*S. trilobata*	y = 3.3722-0.0029x-0.0002x^2^	−7.3	3.4	0.54	21.64	<0.0001
	Invasives	y = 2.5329-0.049x-0.0007x^2^	35.0	3.4	0.53	21.05	<0.0001
	Natives	y = 0.4863+0.1419x-0.0015x^2^	47.3	3.8	0.62	30.44	<0.0001

Note: (1) *H*′ = -Σ[(*P*i) ln(*P*i)]; *D*′ = 1/Σ(*P*i)2; *J*′ = *H*′/(ln*S*). *P*_i_ is the ratio of the cover of species i to the total coverage of all species in each plot; *S* is the number of species in each plot.

(2) Invasives – all invasive plants, Natives - native plants.

**Figure 4:**
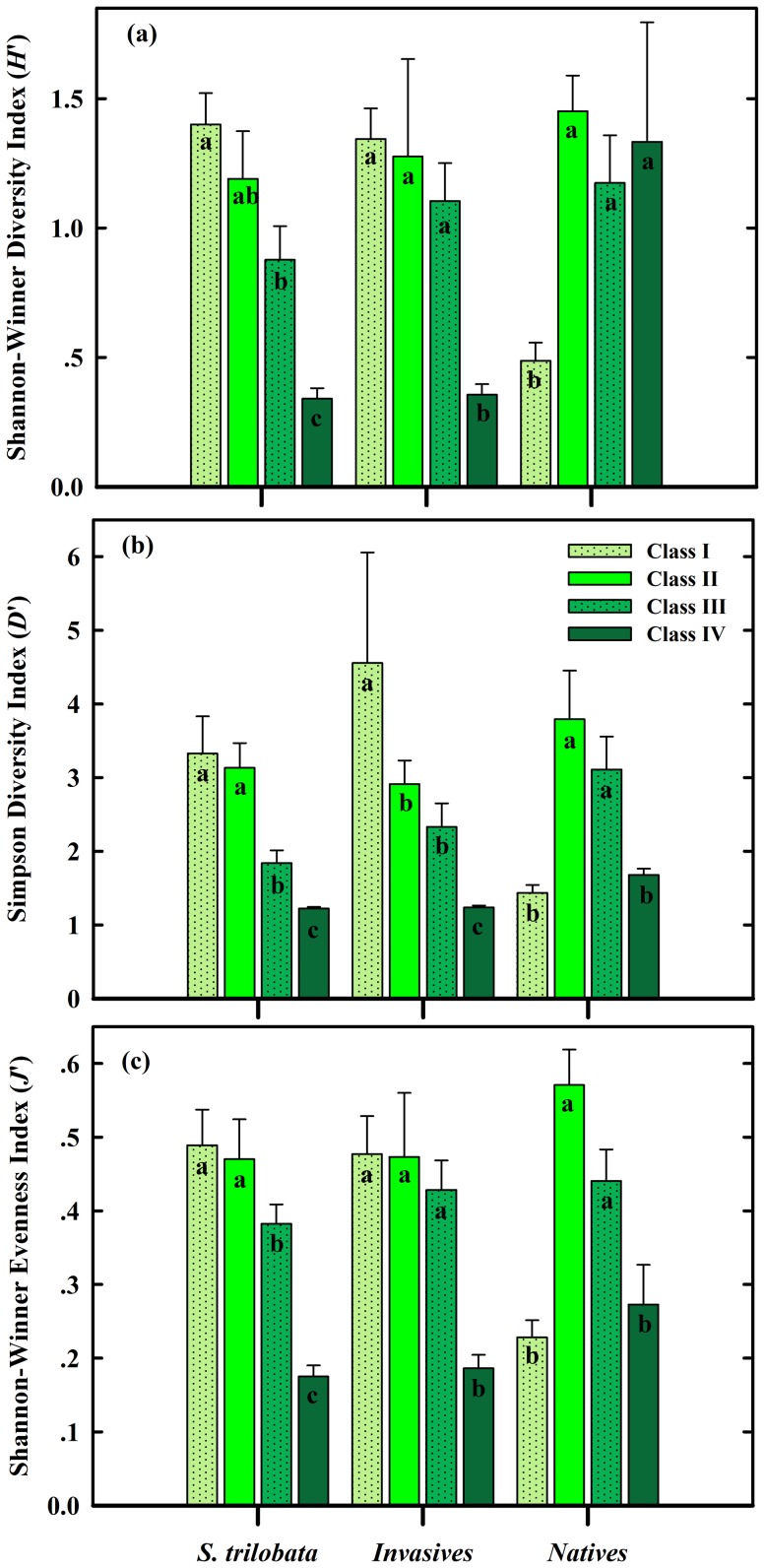
Mean biodiversity of the plant communities among four classes of cover (I, II, III, IV) within the community composition (*S. trilobata*, all invasive plants, and all native plants). (a): Shannon-Wiener's species diversity index (*H′*); (b): Simpson's dominance index (*D′*); (c): Shannon-Wiener's evenness index (*J*′). For each variable, means labeled with the same letter are not significantly different according to Duncan's multiple-range test at the *p* = 0.05 level.

The values of diversity indices were altered by both the sampling cities and the grade of coverage within the community composition (*S. trilobata*, all invasive plants and all native plants) (Table 2). Accordingly, higher grades of coverage of both *S. trilobata* and all invasive plants decreased species diversity (*H*′), species dominance (*D*′), and species evenness (*J*′) in the communities (Fig. 4a-4c). The highest plant diversity appeared under the lowest cover (Grade I) of *S. trilobata* and all invasive plants. However, the lowest and the highest plant diversity appeared under the lowest cover (Grade I) and Grade II cover of native plants, respectively (Fig. 4a-4c).

**Table 2:** Two-way ANOVA for community biodiversity indices (species diversity, dominance, and evenness) among cities, as affected by the cites and four grades of cover within the community composition (*S. trilobata*, all invasive plants and all native plants).

Source of variation	DF	Species diversity index (*H′*)				Community dominance index (*D′*)				Community evenness index (*J′*)			
		SS	MS	*F*	*p*	SS	MS	*F*	*p*	SS	MS	*F*	*p*
City	7	1.55	0.22	4.07	0.0069	13.40	1.91	17.7	<0.0001	0.21	0.03	4.75	0.0032
*S. trilobata*	3	5.49	1.83	33.52	<0.0001	26.19	8.73	80.72	<0.0001	0.39	0.13	20.4	<0.0001
City×*S. trilobata*	10	0.78	0.08	1.42	0.2440	17.17	1.72	15.88	<0.0001	0.08	0.01	1.21	0.3472
Error	19	1.04	0.05			2.05	0.11			0.12	0.01		
Total	39	11.79				66.70				1.23			
City	7	1.76	0.25	4.11	0.0050	16.90	2.41	15.48	<0.0001	0.24	0.03	3.69	0.0087
All invasives	3	5.02	1.67	27.28	<0.0001	31.19	10.40	66.68	<0.0001	0.33	0.11	12.04	<0.0001
City×All invasives	7	0.96	0.14	2.24	0.0698	11.06	1.58	10.14	<0.0001	0.05	0.01	0.83	0.5767
Error	22	1.35	0.06			3.43	0.16			0.20	0.01		
Total	39	11.79				66.70				1.23			
City	7	2.28	0.33	4.2	0.0045	2812.00	401.72	2.56	0.0430	0.15	0.02	1.69	0.1627
All natives	3	4.66	1.55	20.03	<0.0001	9940.80	3313.60	21.15	<0.0001	0.30	0.10	7.94	0.0009
City× All natives	7	1.08	0.15	1.98	0.1037	926.48	132.35	0.84	0.5629	0.02	0.00	0.28	0.9555
Error	22	1.71	0.08			6.11	0.28			0.27	0.01		
Total	39	11.79				66.70				1.23			

### Influence of heterogeneous spatial distribution on species diversity

**Figure 5:**
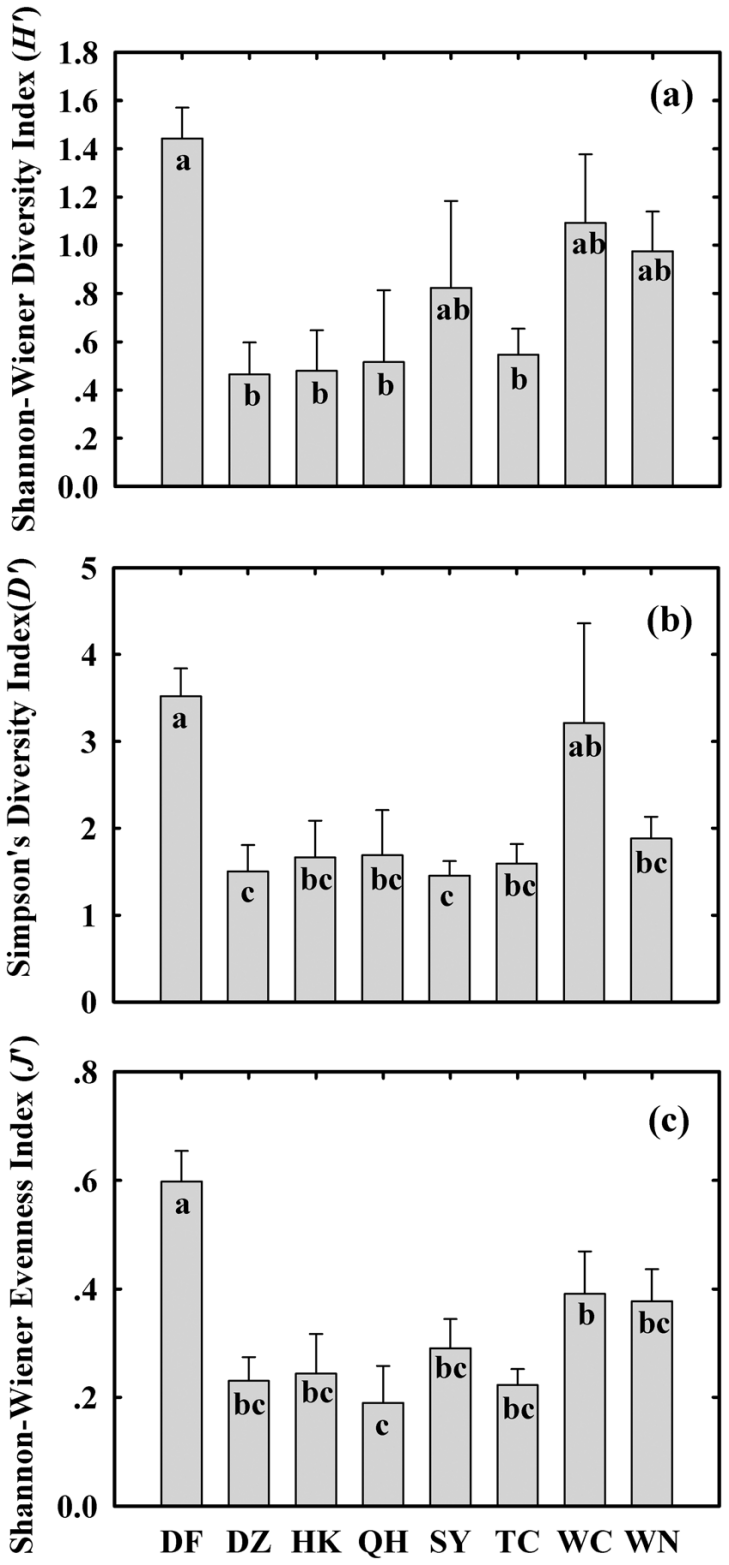
Mean biodiversity of the plant communities among eight cities across Hainan. (a): Shannon-Wiener's species diversity index (*H′*); (b): Simpson's dominance index (*D′*); (c): Shannon-Wiener's evenness index (*J*′). For each variable, means labeled with the same letter are not significantly different according to Duncan's multiple-range test at the *p* = 0.05 level.

There were significant regional differences between the species diversity among these eight cities (Table 2). Dongfang (DF), located on west Hainan Island, presented the highest plant species diversity, whereas DZ, HK, QH and SY showed relatively low plant diversity (Fig. 5).

### Influence of the spatial distribution and local economy on the species composition of plant communities

**Figure 6:**
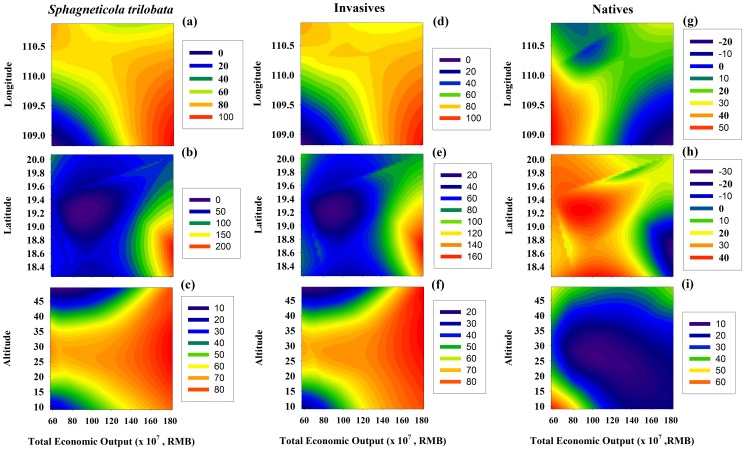
Geographical distribution patterns of the coverage of *S. trilobata* (a, b, c), all invasive plants (d, e, f), and native plants (g, h, i) across different geographical locations (Longitude, Latitude, Altitude) and total economic outputs. The total economic output value included farming, forestry, and animal husbandry in each sampled cities in 2006.

The cover of *S. trilobata* and all invasive plants significantly increased along the examined range of longitudes (Table 3; Fig. 6a, 6d), and the cover of all invasive plants significantly decreased along the altitudinal gradient (Table 3; Fig. 6f).

**Table 3:** Pearson's correlation between soil properties, environmental locations, local economic output and the coverage of *S. trilobata*, invasive plants and native plants.

Factors		*S. trilobata*		Invasives		Natives	
		*r*	*p*	*r*	*p*	*r*	*p*
Location	Longitude	0.4212	**0.0068**	0.4400	**0.0045**	−0.1967	0.2238
	Latitude	0.2332	0.1476	0.2455	0.1268	−0.2122	0.1887
	Altitude	−0.3105	0.0512	−0.3180	**0.0459**	0.2269	0.1591
	Slope	−0.1389	0.3927	−0.0902	0.5800	−0.0049	0.9759
	Hillside	0.1784	0.2707	0.1495	0.3572	−0.0940	0.5642
Economy	Farming	0.1851	0.2529	0.1954	0.2269	−0.1953	0.2273
	Forestry	0.3621	**0.0217**	0.3877	**0.0134**	−0.2884	0.0711
	Grazing	0.3812	**0.0152**	0.3983	**0.0109**	−0.2962	0.0635
	Total output	0.3353	**0.0344**	0.3533	**0.0253**	−0.2875	0.0721

Bold type indicates significant differences at the 0.05 probability level.

A significant positive relationship was found between the economy and the cover of both *S. trilobata* and all invasive species (*r* = 0.34, *p* = 0.034; *r* = 0.35, *p* = 0.025; Table 3). A greater increase in the rate of total economic output in a district corresponded to a higher cover of *S. trilobata* and all invasive species (Fig. 6a-6f) and a lower cover of native species (Fig. 6 g-6i).

## Discussion

### Effects of plants cover on biodiversity

In contrast to previous studies [26], [33], we observed a curvilinear relationship between the cover of invasive/native plants and plant community biodiversity. Many studies have found that invasive plants display a negative linear relationship with species diversity [7], [11]–[13], while others have detected a positive linear relationship [34], [35]. Biodiversity affects ecosystem function and changes along environmental stress gradients [36], which might be due to different underlying factors (e.g., soil fertility), or the presence of scale-dependent effects on diversity due to altering species-area relationships in the invasion of non-native species [1], [37], or the sensitivity of different compositions in communities [17], [33]. The species composition of communities could have far-reaching effects on ecosystem processes [11].

Native species are often considered to be the driving force in increasing biodiversity at local scales [38]. In the present study, the native plants in the communities invaded by *S. trilobata* also contributed to increasing plant diversity when their cover was below 55%. Once their cover exceeded 55%, a negative effect of native plants on local species diversity appeared, possibly due to disequilibrium of communities caused by the rapid growth of some native plants in the sampled plots resulting in homogenization. For example, the percentages of *Eriachne pallescens* cover in plot TC4, *Chrysopogon aciculatus* cover in plot SY4, and *Dendrolobium triangulare* cover in plot SY1 were 87.7%, 92.8%, and 92.8%, respectively.

We also found that the examined biodiversity indices (species diversity, species dominance, and species evenness) did not linearly decrease/increase with increases of *S. trilobata* or all invasive plants. Instead, *S. trilobata* or all invasive plants slightly increased the plant diversity of the invaded community within a certain range of cover values (slight invasion phase), possibly due to direct or indirect facilitation of non-native species during their initial introduction [39]. For example, invasive *Sargassum muticum* increased native species richness at a low percent cover (20%) [33]. This finding is consistent with a study by Melo *et al*. [6] indicating that human disturbance at an intensity below a certain threshold would not trigger irreversible biodiversity loss, and the delivery of ecosystem services would increase up to a point with increasing human disturbance.

However, *S. trilobata* seriously inhibits the growth of most species in an invaded community due to its rapid growth, vegetative reproduction [19], [22] and inhibition of the regeneration of other species [16] via allelochemicals [40]. These effects might also benefit from the clonal integration of *S. trilobata* [19], which can increase the initial spreading of clonal plants into new habitats and thereby alter community structure [41]. Once *S. trilobata* occurs at a low percent cover, its invasion will significantly decrease the plant diversity of invaded communities. *S. trilobata* will cover extensive areas, including roadsides, agricultural and pasture lands, open lots, waste disposal sites, garbage dumps, and other disturbed areas [19], [21]. A similar decreasing community-level species pattern was observed for all invasive plants in the communities invaded by *S. trilobata* within a relatively stable range of percent covers (>35%), in contrast to previous research reporting positive or negative linear effects of invasive plants on biodiversity [26], [33], [39]. These findings suggest that the effects of plant invasion on local biodiversity might show a time lag.

Such time lag effects might be due to the requirement for pre-adaptation to new habitats or could be purely demographic phenomena (e.g., Allee effects) [42]. It is widely recognized that some species present a time lag in their responses to broad-scale land use and land cover changes [4]. There is often a lag phase after non-native species establish small populations before their populations spread [42] because it is necessary for established populations of non-native species to adapt to new habitats (e.g., limiting resources, climates, native competitors, new enemies) and to accumulate a significant population size before the population outbreak [43].

### Effects of human disturbance on the plant distribution

*S. trilobata* is generally used as an ornamental garden species in Hawaii and on other Pacific islands [23] and is universally employed as a greenbelt plant in South China, accompanying anthropogenic transposition of indigenous plants. As a clonal plant, *S. trilobata* rapidly spreads and becomes established in fields once it escapes from gardens [20] because of its noteworthy vegetative dominance over plant communities [19].

Metzger *et al*. [4] found that the landscape history strongly affects the present distribution pattern of species in fragmented landscapes. In addition, Qian *et al.* [12] observed that humans contribute more to the number of exotic species compared with ecological conditions. However, Hainan Island has been subjected to rapid land conversion and loss of the natural forest habitat in the past decades [44], [45], resulting in a decrease in natural forest habitat and increases in farmlands and tourism. Developed regions in Hainan are mostly concentrated in the east and at low altitudes, where human activities are more frequent. Our results also showed that the cover of *S. trilobata* and all invasive plants in the community presented a clear increasing geographical pattern from west to east in Hainan (Table 2-3), consistent with the economic distribution pattern (Table 3; Fig. 6*a, 6d*, 6g). Invasion increases with the intensity of historical land use [25] and economic activities on a global scale [46]. Nevertheless, the abundance of native species might be impacted by habitat loss and the introduction of exotic species [38]. Consequently, local development could promote *S. trilobata* invasion in Hainan via extensively and artificially transforming native species into introduced plants.

Our findings will not only provide managers with urgently required information about the effects of the invasive weed *S. trilobata*, but will also contribute to our broader understanding of the effects of invasive plants on communities.

### Implications of the curvilinear effects of plant invasion

Plant invasion is often positively linked to the intensity of historical land use, which might promote invasion far into the future [25], [47]. Higher levels of community biodiversity increase tolerance to changing environmental conditions and are more beneficial for maintaining ecosystem functions [1], [36]. However, rapid economic development has promoted the expansion of invasive species on a global scale [30]. Global biodiversity, especially for species-rich regions and island ecosystems [8], such as tropical Hainan Island, is suffering unprecedented threats because of large-scale and continuing strengthened economic development and other human activities. As a result of human disturbance, biological invasion has negative impacts on local biodiversity due to altering community structure and/or function dramatically [48]. However, there is a time lag in the ecological consequences of habitat modifications that strongly affect the present distribution patterns of species [4].

Due to the existence of curvilinear effects, introduced non-native species do not present a threat to the local ecological environment in the initial stage. Therefore, insufficient attention is usually paid to these non-native species by concerned governments until the situation is out of control. Thus, prior to the large-scale introduction of exotic horticultural or economic plants, it is necessary to conduct an adequate risk assessment for these introduced plants. Moreover, for introduced exotic species, we recommend that human disturbances should be decreased in local ecosystems, and the eradication of invasive species should be increased to reduce the success of invasion before biodiversity declines.

However, the ecological and the phenological responses of invasive plants to the indicated time-lag effects remain unknown, and further work should be conducted to elucidate the underlying mechanisms of plant invasion. For example, wider ranging surveys involving larger sample numbers should be performed to assess plant invasion, long-term species diversity and the function of community dynamics in permanent plots and for functional comparisons of diversity in invaded communities.

## Supporting Information

Table S1 List of plant species in the investigated plots.
(DOC)
