# Supplementary material for: Curvilinear Effects of Invasive Plants on Plant Diversity: Plant Community Invaded by Sphagneticola trilobata
Source: PLoS One. 2014 Nov 26;9(11):e113964. doi: 10.1371/journal.pone.0113964 (PMC4245253; doi:10.1371/journal.pone.0113964)
Supplement: Table S1 — List of plant species in the investigated plots. (DOC) [file pone.0113964.s001.doc]

| **Table S1.** List of flora species in the investigated community | | | | | | | | | | |  |
| --- | --- | --- | --- | --- | --- | --- | --- | --- | --- | --- | --- |
| **No.** | **Compositions** | **Species** |  | | **No.** | | **Compositions** | | **Species** | |  |
| 1 | Invasive | *Sphagneticola trilobata* |  | | 34 | | Native | | *Buxus sinica* | |  |
| 2 | Invasive | *Eucalyptus tereticornis* |  | | 35 | | Native | | *Duranta repens* | |  |
| 3 | Invasive | *Oxalis corymbosa* |  | | 36 | | Native | | *Dendrolobium triangulare* | |  |
| 4 | Invasive | *Eupatorium odoratum* |  | | 37 | | Native | | *Melia azedarach* | |  |
| 5 | Invasive | *Mimosa pudica* |  | | 38 | | Native | | *Salix babylonica* | |  |
| 6 | Invasive | *Conyza Canadensis* |  | | 39 | | Native | | *Verbenaceae* sp. | |  |
| 7 | Invasive | *Acacia farnesiana* |  | | 40 | | Native | | *Miscanthus* sp. | |  |
| 8 | Invasive | *Mirabilis jalapa* |  | | 41 | | Native | | *Bombax malabaricum* | |  |
| 9 | Invasive | *Passifora foetida* |  | | 42 | | Native | | *Rubiaceae* sp. | |  |
| 10 | Invasive | *Opuntia stricta* |  | | 43 | | Native | | *Kandelia obova* | |  |
| 11 | Invasive | *Lantana camara* |  | | 44 | | Native | | *Fimbristylis tristachya* | |  |
| 12 | Invasive | *Bougainvillea spectabilis* |  | | 45 | | Native | | *Morus alba* | |  |
| 13 | Invasive | *Casuarina equisetifolia* |  | | 46 | | Native | | *Cyperus rotundus* | |  |
| 14 | Alien non-native | *Pandanus tectorius* |  | | 47 | | Native | | *Alchornea davidii* | |  |
| 15 | Alien non-native | *Phyllanthus urinaria* |  | | 48 | | Native | | *Acacia confusa* | |  |
| 16 | Alien non-native | *Ficus elasica* |  | | 49 | | Native | | *Rhodomyrtus tomentosa* | |  |
| 17 | Alien non-native | *Rhoeo discolor* |  | | 50 | | Native | | *Achyranthes aspera* | |  |
| 18 | Alien non-native | *Atropa belladonna* |  | | 51 | | Native | | *Syzygium hainanense* | |  |
| 19 | Alien non-native | *Impatiens balsamina* |  | | 52 | | Native | | *Uvaria tonkinensis* | |  |
| 20 | Alien non-native | *Centella asiatica* |  | | 53 | | Native | | *Lygodium scandens* | |  |
| 21 | Alien non-native | *Nerium indicum* |  | | 54 | | Native | | *Convolvulaceae* sp. | |  |
| 22 | Alien non-native | *Acacia mangium* |  | | 55 | | Native | | *Artemisia absinthium* | |  |
| 23 | Alien non-native | *Garcinia mangostana* |  | | 56 | | Native | | *Cocos nucifera* | |  |
| 24 | Native | *Musa basjoo* |  | | 57 | | Native | | *Vernonia cinerea* | |  |
| 25 | Native | *Mallotus apelta* |  | | 58 | | Native | | *Emilia sonchifolia* | |  |
| 26 | Native | *Areca catechu* |  | | 59 | | Native | | *Caryota ochlandra* | |  |
| 27 | Native | *Flacourtia indica* |  | | 60 | | Native | | *Polyscias fruticosa* | |  |
| 28 | Native | *Hedyotis auricularia* L. var. *auricularia* | |  | | 61 | Native | | *Eupatorium japonicum* | |  |
| 29 | Native | *Hibiscus rosa-sinensis* Linn. | |  | | 62 | Native | | *Eriachne pallescens* | |  |
| 30 | Native | *Nelumbo nucifera* | |  | | 63 | Native | | *Sida corylifolia* | |  |
| 31 | Native | *Ipomoea pes-caprae* | |  | | 64 | Native | | *Chrysopogon aciculatus* | |  |
| 32 | Native | *Lagenaria siceraria* | |  | | 65 | Native | | *Uvaria macrophylla* | |  |
| 33 | Native | *Cyclosorus parasiticus* | |  | |  |  | |  | |  |
|  |  |  | |  | |  | |  | |  | |
